# Investigation of Color and Mechanical Properties of Parts Printed on 3D Printers After Salt Spray Testing

**DOI:** 10.3390/polym17141902

**Published:** 2025-07-09

**Authors:** İsmet Onur Ünal, Oğuz Koçar, Vahap Neccaroğlu, Erhan Baysal, Nergizhan Anaç

**Affiliations:** 1 Department of Mechanical Engineering, Faculty of Engineering, Zonguldak Bülent Ecevit University, Zonguldak 67100, Türkiye; onurunal0013@gmail.com (İ.O.Ü.); nergizhan.kavak@beun.edu.tr (N.A.); 2 Department of Mechanical Engineering, Faculty of Engineering, Bartın University, Bartın 74100, Türkiye; vahapneccaroglu@gmail.com; 3 Alaplı Vocational School, Zonguldak Bülent Ecevit University, Zonguldak 67850, Türkiye; erhanbaysal@beun.edu.tr

**Keywords:** additive manufacturing, 3D printing, aging, salt spray testing, ABS+, mechanical properties

## Abstract

The use of plastic materials in the maritime industry is increasing day by day. Plastics are particularly preferred in watercraft due to their lightweight, resistance to water-related damage (such as mold and wear), optical clarity, and high corrosion resistance. In recent years, plastics produced by 3D printing have gained prominence in applications traditionally dominated by conventional plastic materials. Therefore, producing marine-grade materials—such as acrylonitrile butadiene styrene (ABS), which has long been used in the maritime sector—through 3D printing, and understanding their long-term performance, has become increasingly important. In this study, the mechanical behavior, surface roughness, and color changes of ABS+ materials in three different colors (yellow, green, and blue) and with three different infill ratios (50%, 75%, and 100%) were investigated after a salt spray test. Following the salt spray exposure, tensile and bending tests, hardness measurements, surface roughness analyses, and color measurements were conducted and compared with reference samples. The results were evaluated based on filament color and infill ratio. This study underscores the importance of color selection—along with mechanical strength—when designing 3D-printed materials for long-term use in saltwater environments.

## 1. Introduction

Additive manufacturing (AM) encompasses a wide range of technologies, including material extrusion (MEX), laminated object manufacturing (LOM), stereolithography (SLA), and selective laser sintering (SLS), each tailored for specific material classes and applications [1]. Among these, material extrusion—most commonly represented by Fused Filament Fabrication (FFF), also known as Fused Deposition Modeling (FDM)—has emerged as the most widely adopted AM technique for polymer-based materials [2,3]. This popularity is primarily attributed to its cost-effectiveness, accessibility, and versatility in processing thermoplastics such as acrylonitrile butadiene styrene (ABS), polylactic acid (PLA), polyethylene terephthalate glycol (PETG), and fiber-reinforced composites [4,5]. These polymeric materials offer significant potential for various applications due to their favorable mechanical properties. However, their long-term performance can be adversely affected by environmental stressors such as humidity, ultraviolet (UV) radiation, and thermal cycling [6,7,8]. For polymeric materials, particularly those used in outdoor environments, durability is critically important to ensure both quality and safety, as these materials are exposed to various environmental factors such as temperature fluctuations, humidity changes, and ultraviolet (UV) radiation [9,10,11]. The degradation of polymers under such conditions is known to affect not only their mechanical properties but also their optical characteristics.

In FFF-printed polymer objects, both mechanical and optical properties are influenced by several parameters, including infill ratio, layer thickness, printing temperature, printing speed, filament color, and operating conditions (e.g., UV exposure, humidity, and extreme temperatures) [12,13,14,15,16]. Polymer degradation is typically manifested through a range of physical and chemical changes, such as color fading or discoloration, decreased hardness, and increased surface roughness [17,18].

To understand the mechanisms behind degradation processes, both natural and artificial aging methods are commonly employed. However, a limited number of studies in the literature have investigated the aging behavior of 3D-printed materials [19]. Amza et al. [20] examined the aging of 3D-printed PLA and PETG polymers under 24 h of UV-B radiation and observed a reduction in the mechanical properties of both polylactic acid (PLA) and polyethylene terephthalate glycol (PETG) parts following the aging process. Additionally, they found that PLA samples became shinier, while PETG samples developed a yellow tint. Osadolor et al. [21] exposed PLA samples to UV radiation at 45 °C and 65% relative humidity for durations of 4, 6, and 8 weeks. Their study revealed that increased UV exposure led to a notable enhancement in mechanical properties, particularly in Young’s modulus and compressive strength. In another study, Amza et al. investigated 3D-printed ABS-PC copolymer samples (a blend of acrylonitrile butadiene styrene and polycarbonate) exposed to UV-C and UV-B light for 24 h. They observed that the color change in the copolymer was consistent with the degradation of the ABS component, and a decrease in mechanical properties was detected after aging. Nevertheless, artificial aging tests have certain limitations, as they cannot fully replicate natural environmental conditions [22]. For instance, Santos et al. [6] found that ABS polymers exhibited a greater level of degradation under artificial aging conditions compared to those exposed to the natural Mediterranean climate. This finding highlights the limitations of artificial aging in accurately predicting long-term performance.

The literature review reveals that the changes in the mechanical properties of engineering plastics—such as ABS, which is commonly used as a filament material in 3D printing—before and after aging have not been sufficiently investigated. Among polymeric materials, ABS stands out due to its high mechanical strength and chemical resistance [23]. It is widely used in critical sectors such as automotive, marine, military, aerospace, and consumer electronics [19,24]. To evaluate the suitability of ABS products for marine environments, aging tests such as salt fog or salt spray testing are commonly employed [25]. These tests assess the effects of saltwater exposure on the mechanical performance of materials. Moreno Nieto et al. examined the water absorption and degradation behavior of PETG and PLA materials fabricated using FFF technology. Their study found that increasing the infill ratio resulted in reduced water absorption and improved mechanical strength [26]. Furthermore, surface properties such as roughness and gloss significantly influence both the functional and esthetic performance of the material [27]. Exposure to UV radiation can negatively impact the color stability and mechanical integrity of ABS [20]. In their study on ABS and its composites, Iannuzzi et al. reported that both natural and accelerated aging led to noticeable color fading and increased hardness [28]. Similarly, Gao et al. tested ABS and PLA materials in eight different colors and found that filament color had a significant effect on the mechanical properties of 3D-printed components [12]. Despite these findings, comprehensive studies that examine the combined effects of infill ratio and material color on the post-aging behavior of FFF-printed ABS+ are still limited. This highlights a critical research gap in understanding polymer durability in additive manufacturing.

This study aims to investigate the effects of aging on ABS+ materials characterized by three different infill ratios (100%, 75%, and 50%) and three different colors (yellow, green, and blue). The research focuses on evaluating surface roughness, mechanical properties (tensile strength, flexural strength, and hardness), and color changes. The results of this study are expected to provide valuable insights into the effects of artificial aging on additively manufactured materials and contribute to performance optimization in polymer-based AM applications.

## 2. Material and Method

### 2.1. Material Properties

In the experiments, ABS+ filament produced by eSUN was used. ABS is inherently a rigid material, known for its high resistance to impact and scratching, as well as its strong tensile and impact strength. Additionally, it is recyclable. Owing to these characteristics, ABS is suitable for general-purpose applications and is commonly preferred in products requiring durability against impact and heat.

ABS+, on the other hand, is a modified version of standard ABS with enhanced mechanical properties. Specifically, ABS+ offers approximately 40% higher tensile strength, 20% greater flexural strength, and lower moisture absorption compared to conventional ABS [29]. Due to these advantages, ABS+ was selected as the material for this study. The material properties provided by the manufacturer are presented in Table 1.

### 2.2. Three-Dimensional Printing Parameters

**Figure 1:**
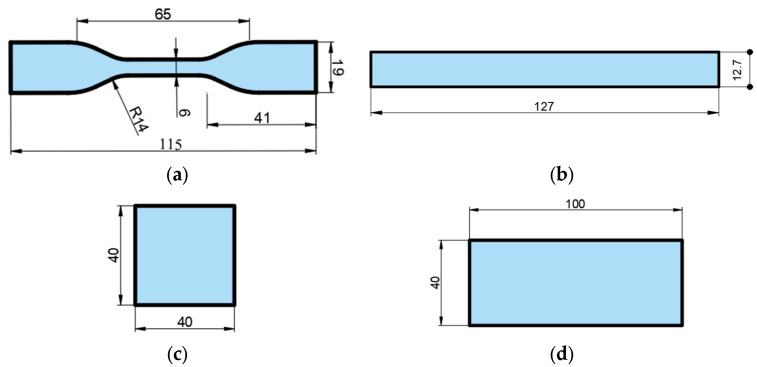
Dimensions of samples used in the study: (**a**) tensile, (**b**) bending, (**c**) hardness, and (**d**) surface roughness and color measurement samples.

To determine the mechanical properties of the samples used in this study, tensile test specimens were prepared in accordance with ASTM D638 Type IV [31], while three-point bending test specimens were fabricated according to ASTM D790 [32]. For color and surface roughness measurements, rectangular plates with dimensions of 100 × 40 × 4 mm were printed. Additionally, square plates measuring 40 × 40 × 4 mm were produced for hardness measurements (Figure 1). All samples were manufactured using the Fused Filament Fabrication (FFF) method with an Ultimaker S5 3D printer. Specimens were produced in three different infill ratios (100%, 75%, and 50%) and three different colors (yellow, green, and blue), using a nozzle diameter of 0.4 mm. During the printing process, the nozzle temperature was set to 245 °C, the bed temperature to 90 °C, and the printing speed to 45 mm/s.

**Figure 2:**
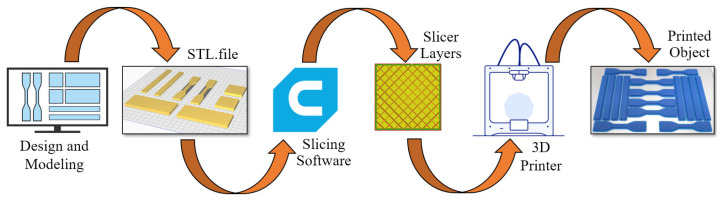
Methodology flow chart for 3D printing.

The printed parts were divided into two groups: reference samples and aged samples. The G-code files required for the printing process were generated using the slicing software “Ultimaker Cura 5.7.0” and then transferred to the printer. A flowchart illustrating the 3D printing process is presented in Figure 2.

### 2.3. Aging Process

Currently, there is no internationally recognized standard that defines the conditions for hydrothermal aging tests of plastic materials [23]. In the literature, hydrothermal aging durations vary widely—from 24 h to 1344 h—depending on the research objectives and the types of materials examined [33,34,35]. While previous studies have typically focused on the effects of aging duration with fixed material parameters, this study investigates the impact of hydrothermal aging on the mechanical properties of ABS+ materials with varying colors and infill ratios. For hydrothermal aging, the samples were subjected to a salt spray test (humid heat and salt fog) at 35 °C for 96 h. The salt spray test is commonly used to simulate marine environments and evaluate the effects of saltwater exposure on materials. In this study, a Precision Salt Spraying Tester Machine (Dongguan Hongtua Instrument Co., Ltd., Dongguan, China) was used as the salt spray chamber. The device has an internal volume of 108 L, with internal dimensions of 600 × 400 × 400 mm, and operates within a temperature range of 35 °C to 50 °C ± 1 °C. For the test, a solution consisting of 5% sodium chloride in distilled water was prepared in accordance with standard procedures.

### 2.4. Tests Performed (Tensile and Bending Tests, Hardness, Color Measurement)

Tensile tests were conducted in accordance with ASTM D638 (Figure 1a), and three-point bending tests were performed in accordance with ASTM D790 (Figure 1b) for the samples with different colors and infill ratios used in this study. Both tests were carried out using a WDW-5 universal testing machine with a capacity of 5 kN. The testing speed was set to 2 mm/min, and all tests were conducted at room temperature, with four repetitions for each sample to ensure statistical reliability. Hardness measurements were performed according to ASTM D2240 [36] using a Loyka Shore-D durometer (Shenzen Yibai Network Technology Co. Ltd., Shenzhen, China). For each sample, hardness was measured at ten different points, and the average value was calculated. The dimensions of the samples used for hardness tests are shown in Figure 1c, while those used for surface roughness and color measurements are shown in Figure 1d. Color measurements were carried out using a CHNSpec CS410 spectrocolorimeter (Hangzhou, China), which provides L*, a*, and b* values based on the CIELAB color space. The measurements were conducted in accordance with ASTM D2244 [37], with four measurements taken per sample. The average of these values was used to determine the final color measurement for each specimen. For surface roughness evaluation, a Mitutoyo SJ301 portable surface roughness tester (Kawasaki, Japan) was used. Five repeated measurements were taken on each sample, and the average surface roughness value was recorded.

## 3. Findings and Discussion

**Figure 3:**
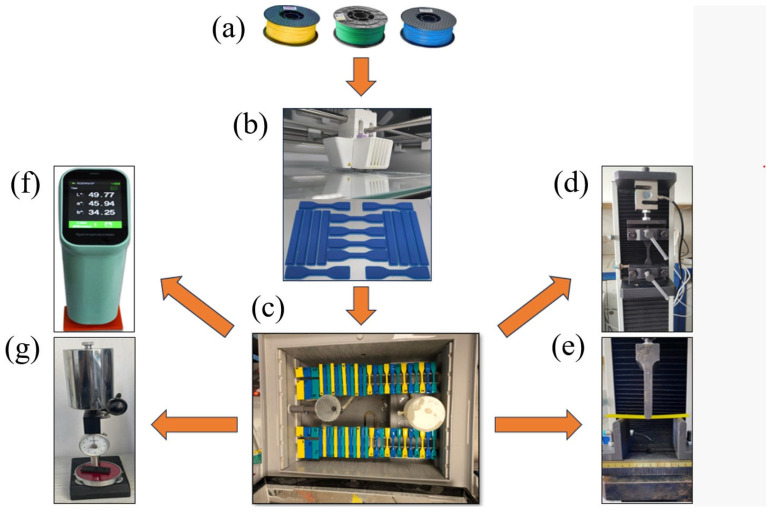
Three-dimensional printing, aging, and testing stages: (**a**) ABS+ filament, (**b**) Ultimaker S5 3D printer, (**c**) aging process, (**d**) tensile testing, (**e**) bending testing, (**f**) color measurement, (**g**) hardness measurement.

In the experiments, yellow, green, and blue ABS+ filaments were used, and samples for each color were prepared with three different infill ratios (100%, 75%, and 50%) using an Ultimaker S5 printer (Ultimaker, Utrecht, Netherlands). The samples were divided into two groups: reference and aged. Mechanical tests and color measurements were conducted separately for both groups. Figure 3 presents a flowchart illustrating the printing and aging procedures. Specifically, Figure 3a shows the filament materials, while Figure 3b depicts the 3D printer during the printing process. Figure 3c illustrates the arrangement of the samples inside the salt spray test chamber. Figure 3d–g display the test setups for tensile, bending, color, and hardness measurements, respectively.

### 3.1. Mechanical Properties of Reference Samples

#### 3.1.1. Tensile Strength Results

**Figure 4:**
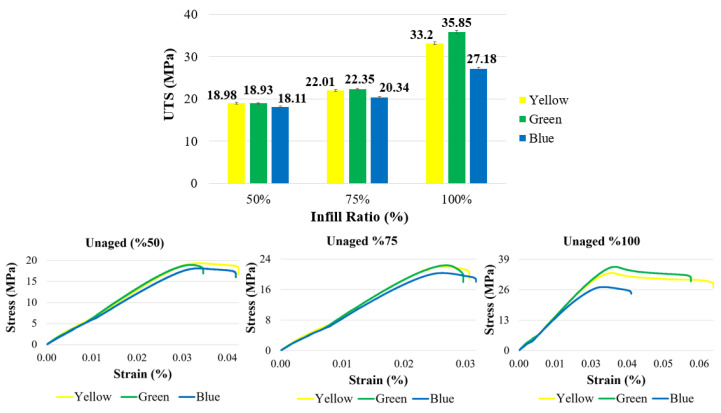
Tensile strength and stress–strain graphs of reference materials according to infill ratio.

The tensile strengths and stress–strain curves of ABS+ samples with different infill ratios and colors are presented in Figure 4, while the ultimate tensile strength (UTS) and elongation values are summarized in Table 2. When compared based on infill ratio, the highest tensile strength values for all three colors (yellow, green, and blue) were observed at 100% infill ratio. Increasing the infill ratio reduces the number of internal voids within the structure. As the cross-sectional area carrying the tensile load increases, the tensile strength of the samples also improves [38,39]. At 100% infill ratio, the highest tensile strength was measured in the green specimens, with a value of 35.85 MPa. The yellow and blue samples exhibited tensile strengths of 33.2 MPa and 27.18 MPa, respectively. This difference may be attributed to the influence of color additives used in the filaments, which can affect the mechanical properties of the material. Examining the elongation values in Table 2, the green and yellow samples showed similar elongation ratios, whereas the blue specimens exhibited lower elongation. In a study conducted by Gao et al. [12] using eight different colors (red, yellow, blue, green, orange, purple, black, and natural) of eSUN ABS filament, tensile strength ranged between 18.8 and 24.9 MPa, and flexural strength between 32.5 and 35.9 MPa. Among these, the red samples showed the highest tensile strength, while the green ones exhibited the highest flexural strength. The tensile strength values obtained in the present study are consistent with those reported in the literature.

As shown in Figure 3, among the samples with a 50% infill ratio, the lowest tensile strength (18.11 MPa) was observed in the blue-colored samples. Compared to the blue samples, the green samples exhibited an increase of approximately 4.5% (18.93 MPa), while the yellow samples showed a 4.8% increase (18.98 MPa). At a 75% infill ratio, the lowest tensile strength (20.34 MPa) was again observed in the blue samples, similar to the 50% infill group. Compared to the blue samples, the green samples demonstrated a 9.9% increase (22.35 MPa), and the yellow samples showed an 8.2% increase (22.01 MPa).

**Figure 5:**
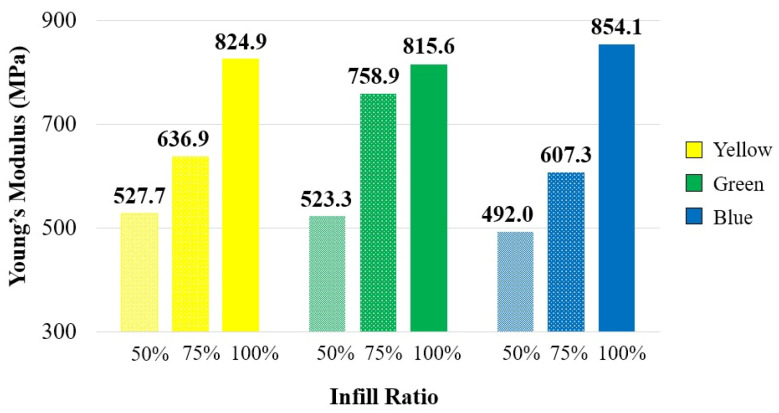
Young’s modulus of reference specimens.

Figure 5 presents the variation in Young’s modulus as a function of color and infill ratio. The study by Kaygusuz and Özerinç investigated the effects of nozzle temperature (190, 200, 210, and 215 °C) and infill ratio (10–100%) on the mechanical properties of PLA materials with varying infill ratios. Their results indicated that Young’s modulus was not significantly affected by nozzle temperature; however, it showed considerable variation with changes in infill ratio [40]. Consistent with these findings, the current study observed an increase in Young’s modulus with higher infill ratios. At infill ratios of 50% and 75%, the yellow specimens exhibited the highest moduli of elasticity, measuring 721.9 MPa and 941.4 MPa, respectively. Conversely, the green specimens achieved the highest modulus at 100% infill ratio, displaying a value of 1171.2 MPa. Across all infill ratios tested, the blue specimens consistently demonstrated a lower modulus of elasticity compared to the other color variants.

**Figure 6:**
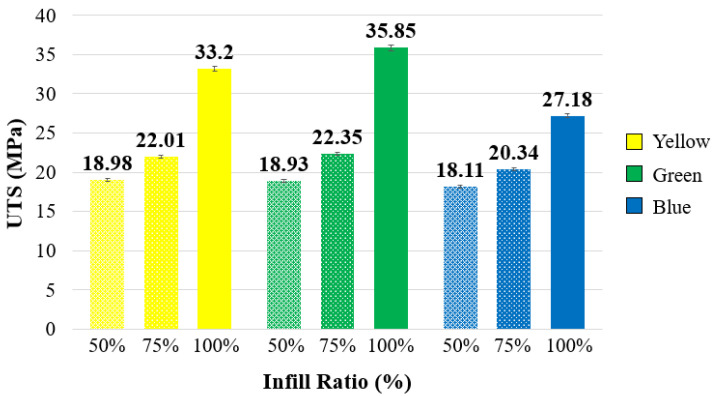
UTS values in reference materials by color.

In general, at 50% and 75% infill ratios, the yellow and green samples exhibited similar performance, whereas at 100% infill, the green samples demonstrated superior tensile strength. These results indicate that the mechanical properties of parts printed in different colors vary depending on the infill ratio. A study involving orange and black samples similarly reported that tensile strength performance varied with both color and infill ratio [41]. Moreover, as shown in Figure 6, strength values increased with increasing infill ratio for all colors. While no significant difference was observed between 50% and 75% infill ratios, a notable increase in tensile strength was recorded at the 100% infill ratio.

**Figure 7:**
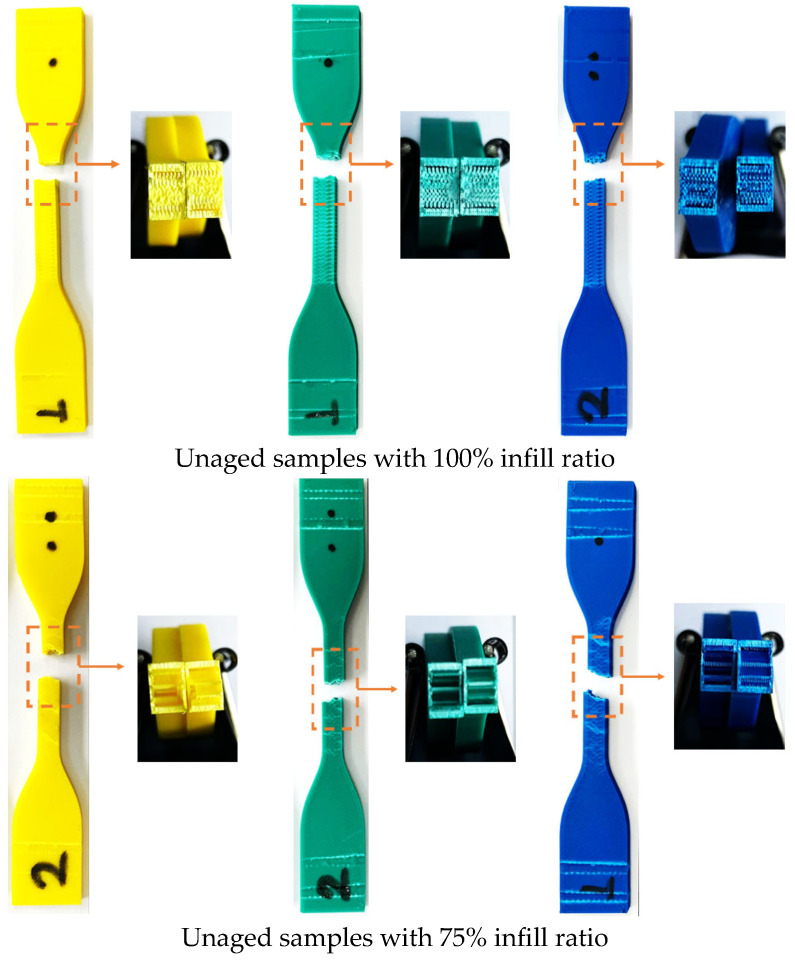

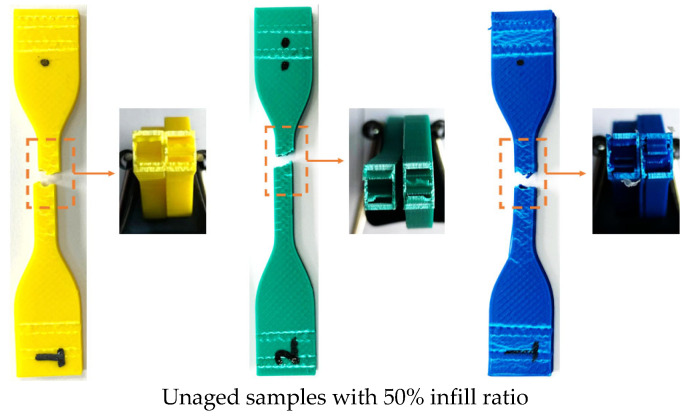
Fracture images of unaged samples after tensile testing for all infill ratios.

The fracture appearances of the unaged test samples after tensile testing are shown in Figure 7. The fracture surface morphology of the parts with a 100% infill ratio differs from those with 50% and 75% infill ratios. In samples with 50% and 75% infill ratios, the fracture patterns were similar, and fractures followed the patterned internal structure of the part. In contrast, for samples with a 100% infill ratio, the fracture occurred in the relatively narrow gauge section just below the fixed area.

#### 3.1.2. Bending Results

**Figure 8:**
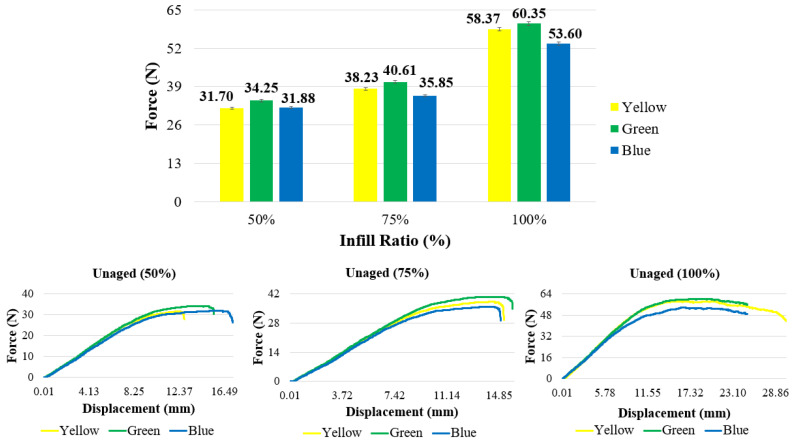
Force–displacement graphs of reference materials.

**Figure 9:**
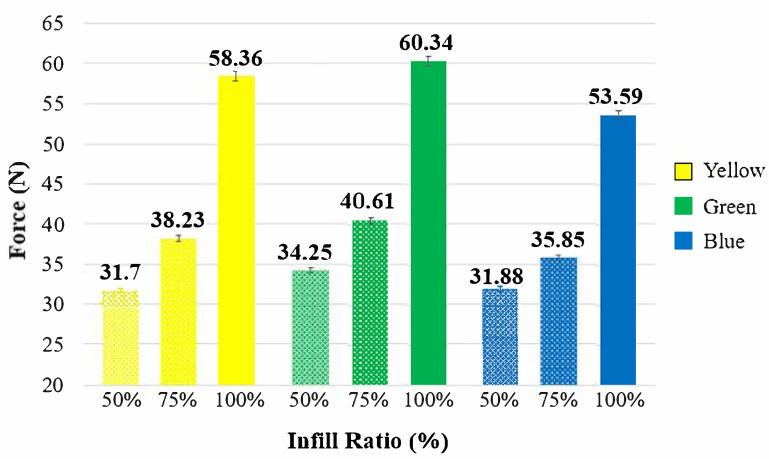
Bending graphs of reference samples.

Figure 8 and Table 3 present the maximum bending forces and force–displacement curves of the samples according to different colors and infill ratios. For all colors, the bending force increased with increasing infill ratio (Figure 9). At all infill ratios, the highest bending forces—60.35 N, 40.61 N, and 34.25 N—were recorded for the green-colored samples. At a 50% infill ratio, the lowest bending force (31.70 N) was observed in the yellow samples, while at 75% infill ratio, the lowest value (35.85 N) was recorded in the blue samples.

**Figure 10:**
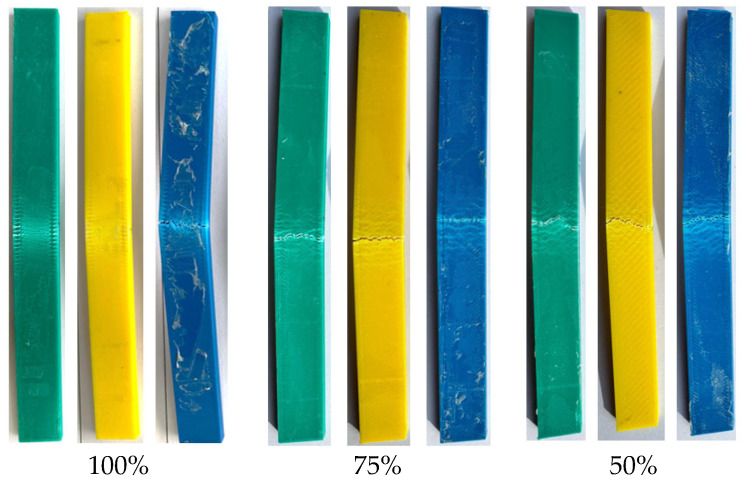
Surface images after bending test.

In the reference samples at a 50% infill ratio, the lowest bending force (31.70 N) was observed in the yellow-colored samples, while the green and blue samples exhibited bending forces of 34.25 N and 31.88 N, respectively. At a 75% infill ratio, the lowest bending force (35.85 N) was recorded in the blue samples. Compared to the blue samples, the green samples showed a 13.3% increase in bending force (40.61 N), and the yellow samples exhibited a 6.6% increase (38.23 N). At a 100% infill ratio, the lowest bending force (53.6 N) again occurred in the blue samples, while the green and yellow samples showed increases of 12.6% (60.35 N) and 8.9% (58.37 N), respectively. The fracture appearances of the unaged test samples after the bending test are shown in Figure 10. The fracture paths observed in the samples with 50%, 75%, and 100% infill ratios were similar to each other.

#### 3.1.3. Hardness of Reference Specimens

**Figure 11:**
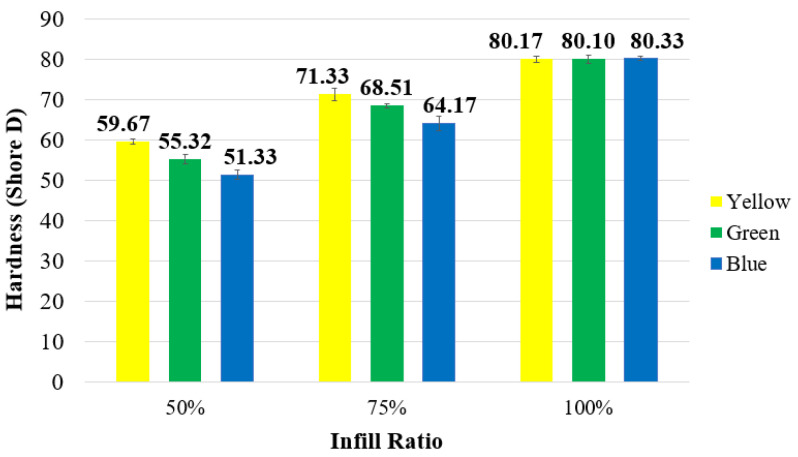
Unaged Shore D hardness values.

Figure 11 presents the Shore D hardness values of the samples according to different colors and infill ratios. At a 100% infill ratio, hardness values were very similar across all colors, indicating that color did not have a significant effect on hardness. However, a decrease in infill ratio negatively affected the hardness values. The greatest reduction in hardness, approximately 36%, was observed in the blue samples, while the smallest decrease, about 25.5%, was recorded in the yellow samples.

The hardness values of fully dense materials (100% infill) were found to be nearly identical, as shown in Figure 11. Since these samples lack internal voids, the observed similarity in hardness is expected and suggests no significant correlation between filament color and hardness.

Conversely, in samples with 50% and 75% infill ratios, which contain porous structures, hardness values exhibited notable variation. However, attributing this variation solely to the influence of color additives is challenging. The experimental results demonstrate that hardness decreases with decreasing infill ratios, regardless of color. Similar trends have been documented in previous studies [42,43,44].

#### 3.1.4. Surface Roughness of Reference Specimens

**Figure 12:**
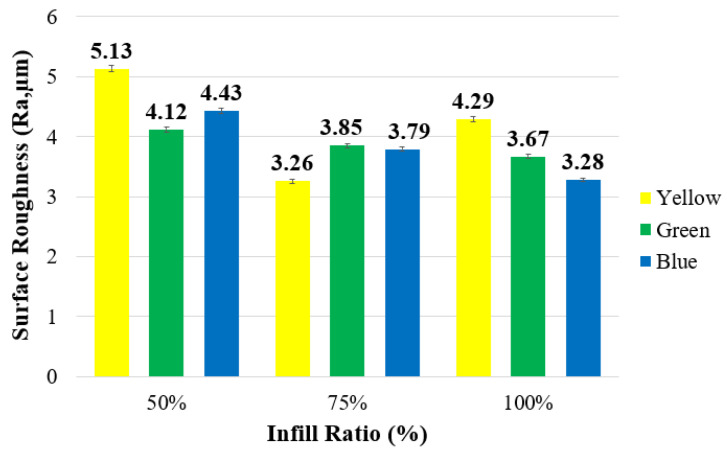
Surface roughness values depending on color in parts with different infill ratios.

The surface roughness (Ra) values of the samples with different infill ratios and colors are presented in Figure 12. At a 100% infill ratio, the surface roughness values, ranked from highest to lowest, were 4.29 μm for yellow, 3.67 μm for green, and 3.28 μm for blue samples. At a 75% infill ratio, a decrease in surface roughness was observed only in the yellow samples. Among all infill ratios, the lowest surface roughness was measured in the yellow samples at 75% infill ratio, with a value of 3.26 μm, while the highest surface roughness of 5.13 μm was recorded in the yellow samples at 50% infill ratio.

The surface roughness of a printed part is influenced by several factors, including the vibration, optimization of process parameters [45,46], material composition [47], additives, pigments, and other variables. During the printing of parts with 100% infill ratio, minimal vibration is observed, whereas lower infill ratios are associated with increased vibration levels. This phenomenon can be attributed to mechanical oscillations generated as the print head moves across the infill pattern. In fully dense parts (100% infill), the absence of internal patterning significantly reduces vibration. Previous studies have confirmed that vibration remains consistent at full infill but varies with decreasing infill ratio [48]. Additionally, fully dense parts exhibit greater rigidity [49]. As a result, 100% infill leads to superior surface quality due to enhanced structural stability, while infill ratios of 75% and 50% contribute to progressively poorer surface finishes.

In the case of yellow specimens with 75% infill, the variation in surface quality may be attributed to the coloring agent used. However, previous studies have not identified a conclusive explanation for the influence of filament color on surface roughness. It is known that pigments exhibit distinct wetting properties; insufficient wetting can result in slow and ineffective dispersion, pigment sedimentation, increased viscosity, and impaired flow behavior. The flow resistance of a liquid increases with viscosity, and it is well-established that pigments contribute to elevated viscosity, with their effects directly correlated to particle size. Nevertheless, the challenge of determining the type or concentration of color pigments in commercially available filaments complicates establishing a definitive relationship between color and resulting properties such as surface roughness.

### 3.2. Aged Samples

#### 3.2.1. Tensile Test Results of Aged Specimens

**Figure 13:**
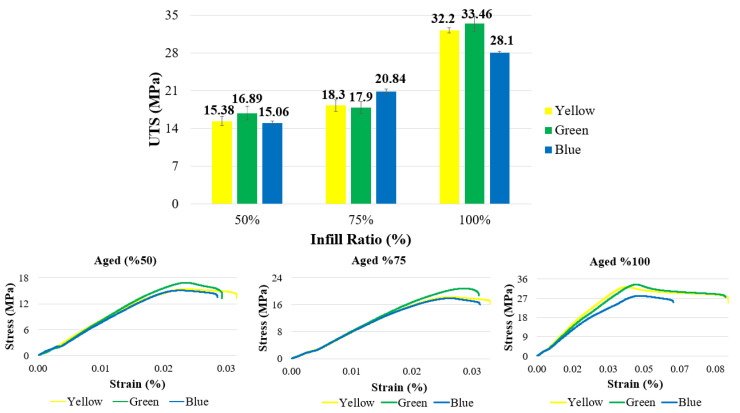
Tensile strength and stress–strain graphs of aged materials.

The ultimate tensile strength (UTS) values and stress–strain curves of the aged samples after tensile testing are presented in Figure 13 and Table 4. For samples with a 100% infill ratio, the tensile strengths were recorded as 33.46 MPa for green, 32.2 MPa for yellow, and 28.1 MPa for blue samples. The highest tensile strength was observed in the green samples at both 50% and 100% infill ratios, whereas at 75% infill ratio, the blue samples exhibited the highest value. Across all infill ratios, the yellow samples showed the greatest percentage elongation.

**Figure 14:**
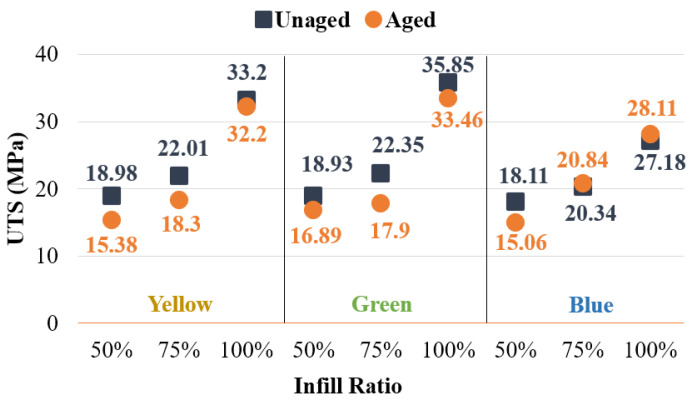
Comparison of tensile strengths of unaged and aged samples.

As shown in Figure 14, compared to the unaged (reference) samples, the tensile strength of the green samples with a 100% infill ratio decreased by 7.1% after aging, while the yellow samples showed a 3.1% decrease, and the blue samples exhibited a 3.4% increase. At 100% infill ratio, aging negatively affected the green and yellow samples, whereas the blue samples demonstrated an improvement in tensile strength. This unexpected improvement is attributed to the phenomenon that 3D-printed components exhibit reduced degradation following aging compared to their conventionally manufactured counterparts [35]. Numerous studies in the literature support the enhancement of mechanical properties in polymer materials after aging [23,50,51,52,53,54]. At a 75% infill ratio, compared to the reference samples, the green samples showed a 24.9% decrease in tensile strength, the yellow samples a 20.2% decrease, and the blue samples a 2.5% increase after aging.

When the infill ratio was 50%, tensile strength after aging decreased by 12.1%, 23.4%, and 20.3% for green, yellow, and blue samples, respectively. Notably, an increase in tensile strength was observed in blue specimens at 100% and 75% infill ratios after aging. This indicates that both infill ratio and color are important factors influencing aging behavior.

In a study involving ABS, PLA, PETG, and acrylonitrile styrene acrylate (ASA) filaments, it was reported that UV exposure at 400 mm/20 h caused yellowing in ABS filament and stickiness in PLA filament. Another study subjected ABS and ASA filaments of different brands and colors to an aging process, observing that mechanical properties and color changes after aging varied depending on the filament brand and color. This variation was attributed to the color additives used by manufacturers during filament production [55].

In conclusion, colorants significantly influence both the mechanical properties and post-aging behavior of the material. Mechanical tests conducted on samples of different colors clearly demonstrate the impact of color on material performance. Furthermore, it was observed that in aged green samples, tensile strength values at 50% and 75% infill ratios (16.89 MPa and 17.9 MPa, respectively) were quite similar. Blue samples at 75% and 100% infill ratios, as well as yellow samples at 100% infill ratio, were not significantly affected by aging. These findings suggest that material color should be considered, especially for parts intended for outdoor use, such as in coastal areas, under sunlight, or in cold environments.

Montalvão et al. [54] conducted an investigation in which PLA and PLA-PHA specimens with infill densities of 40% and 80% were immersed in seawater for durations of 15, 30, and 45 days. It was observed that the ultimate tensile strength (UTS) of specimens with 80% infill decreased consistently across all exposure periods. In contrast, specimens with 40% infill exhibited an increase in UTS following 30 days of immersion. For PLA-PHA specimens, an increase in UTS was noted after 15 and 45 days of exposure at 80% infill, while enhancements in UTS were reported for all groups with 40% infill. This behavior was attributed to the stability of PLA’s molecular weight during immersion in seawater at approximately 25 °C, which prevented the scission of polymer chains [56].

Kakanuru and Pochiraju conducted a comparative study on moisture absorption and degradation between biodegradable PLA and non-biodegradable ABS. Samples were immersed in distilled water at 50 °C for 140 days, with periodic weight measurements. The results indicated that the diffusion coefficient of ABS was approximately half that of PLA. Notably, ABS specimens exhibited superior structural integrity in post-desorption testing compared to PLA [51].

Collectively, these studies suggest that ABS is more suitable for marine applications than PLA, particularly in terms of degradation resistance at temperatures ranging from 30 °C to 50 °C. These findings are consistent with our results, which indicate that while aged specimens with 100% infill retained their mechanical properties, performance variations based on color were observed at lower infill densities.

**Figure 15:**
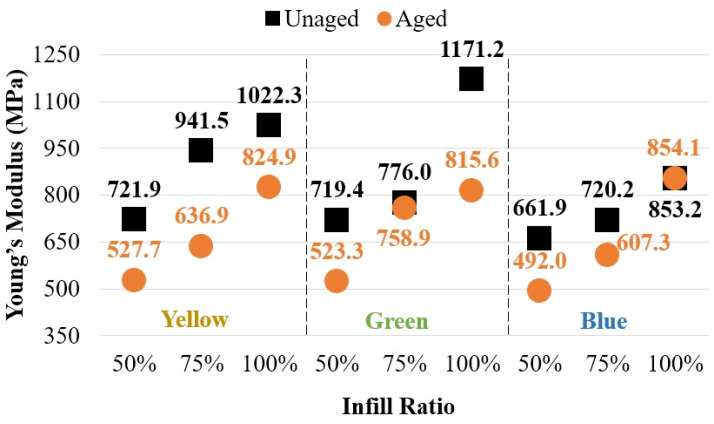
Comparison of elastic modulus values between aged and unaged specimens.

Figure 15 illustrates the elastic modulus values for both unaged and aged specimens. It was observed that the aging process had a minimal effect on the elastic modulus of green specimens at 75% infill and blue specimens at 100% infill. In contrast, significant reductions in elastic modulus were noted in specimens with other color and infill combinations following aging.

**Figure 16:**
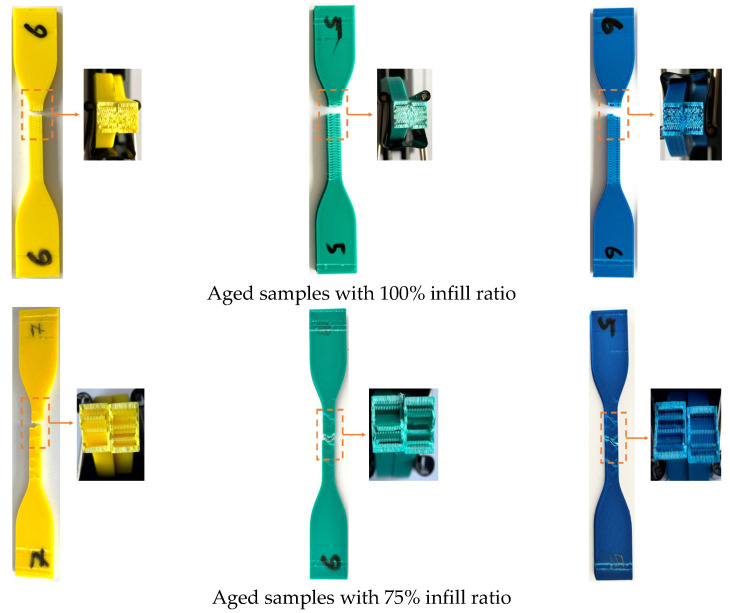

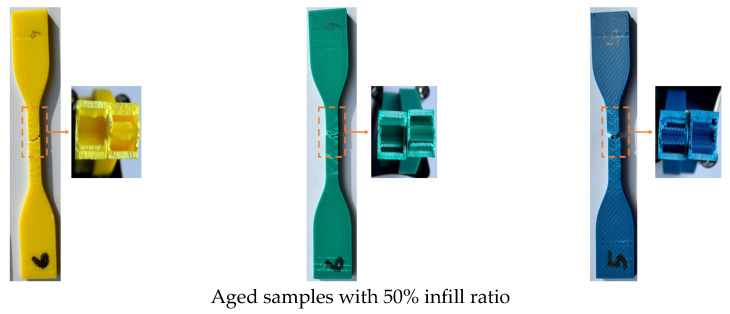
Fracture images of aged samples after tensile testing for all infill ratios.

Figure 16 shows the fracture appearance of the aged test samples after the tensile test. For samples with 50% and 75% infill ratios, the fracture types appeared similar, with damage confined to the gauge length. In specimens with a 100% infill ratio, the fracture surfaces were smooth and exhibited sharp edges. When compared to the fractures in the unaged specimens (Figure 7), it can be observed that the fracture patterns in the aged specimens are similar to those in the unaged specimens, depending on the infill ratio.

#### 3.2.2. Bending Test Result of Aging Specimens

**Figure 17:**
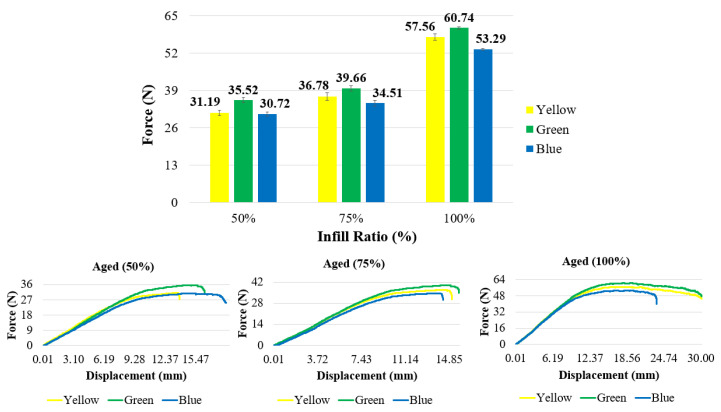
Force–displacement graphs of aged materials.

The flexural force and force–displacement curves after the bending test for all infill ratios of the aged specimens are presented in Figure 17 and Table 5. For all infill ratios, the highest bending forces (60.74 N, 39.66 N, and 35.52 N) were observed in the green-colored samples. In contrast, the lowest bending forces (53.29 N, 34.51 N, and 30.72 N) were recorded in the blue-colored samples across all infill ratios. When examining the displacement distances at which fracture or cracking first occurred under bending load (Table 6), the values were found to be 17.44 mm for the blue samples at 50% infill ratio, 15.79 mm for the green samples at 75% infill ratio, and 32.10 mm for the yellow samples at 100% infill ratio.

**Figure 18:**
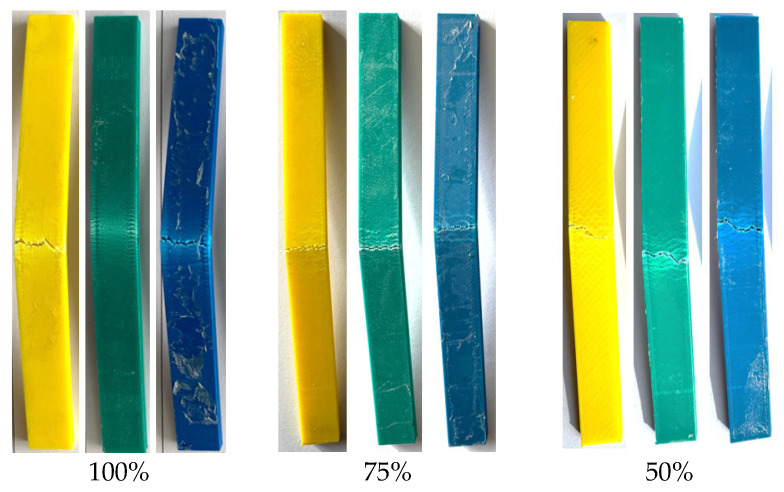
Fracture surfaces of the samples after the bending test.

Figure 18 shows the fracture surfaces of the samples after the bending test. It can be observed that the applied load during the bending test caused cracking along the loading direction. The samples underwent plastic deformation without completely fracturing into two pieces.

**Figure 19:**
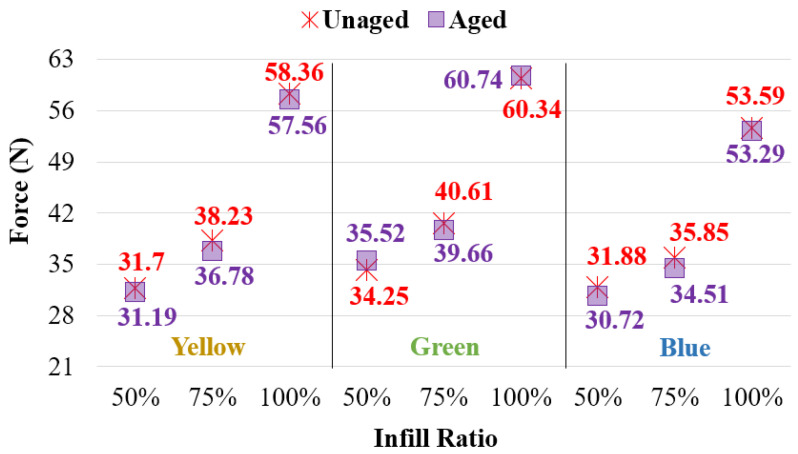
Comparison of bending forces of unaged and aged samples.

Figure 19 presents the bending force graph of the aged and unaged samples. Across all infill ratios, the difference between aged and unaged samples ranged between 0.5% and 4%. No significant change in bending force was observed after aging.

#### 3.2.3. Hardness Results of Aging Specimens

**Figure 20:**
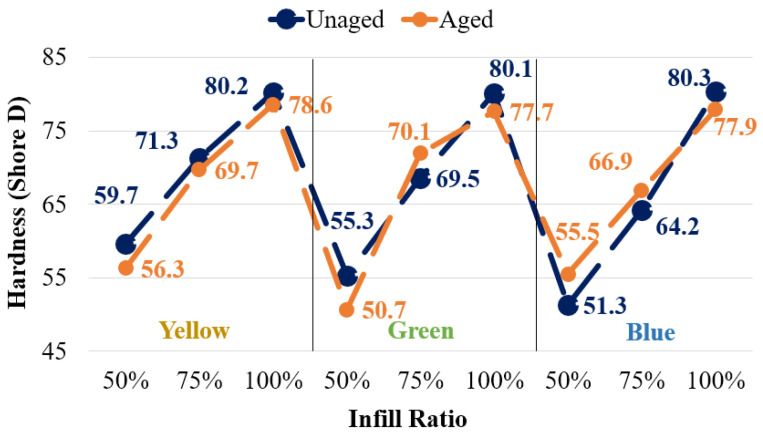
Shore D hardness values of unaged and aged samples.

The Shore D hardness values of the unaged and aged samples with different colors and infill ratios are presented in Figure 20. At a 100% infill ratio, aging resulted in an average hardness decrease of approximately 2% across all colors. However, at 50% and 75% infill ratios, the blue samples exhibited a tendency for increased hardness after aging, whereas a decrease was observed in the yellow samples. The increase in hardness of the blue samples with 75% infill ratio aligns with the higher tensile strength observed in Figure 14. Nevertheless, special attention should be given to the internal voids during hardness measurements at 50% and 75% infill ratios, as they may affect measurement accuracy.

#### 3.2.4. Surface Roughness of Aging Specimens

**Figure 21:**
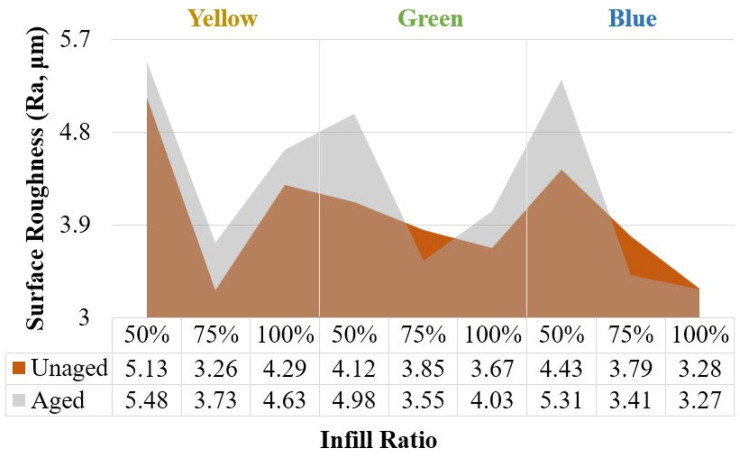
Surface roughness of unaged and aged samples.

Figure 21 presents the surface roughness values measured from the samples before and after aging. The highest surface roughness values before and after aging were recorded in the yellow samples with a 50% infill ratio, measured as 5.13 μm and 5.48 μm, respectively. Surface roughness increased across all infill ratios for the yellow samples. For the green samples, surface roughness was higher at a 50% infill ratio, while it was relatively lower at 75% and 100% infill ratios. The lowest surface roughness before aging was measured as 3.26 μm in the yellow sample at 75% infill ratio, and after aging, the lowest value was 3.27 μm in the blue sample at 100% infill ratio. At 50% infill ratio, surface roughness values increased for all colors, which can be attributed to the porous internal structure of the parts being more susceptible to moisture and heat, leading to surface degradation.

### 3.3. Color Difference After the Aging of Samples

In the experiments, ABS+ materials in three different colors—yellow, green, and blue—were used. To express the color differences of the parts as a single value, the average values of Δa*, Δb*, ΔL*, and ΔE* were calculated using the relevant formulas (Equations (1)–(5)).

(1) $$∆E^{*}=\sqrt{{\left(∆L^{*}\right)}^{2}+{\left(∆a^{*}\right)}^{2}+{\left(∆b^{*}\right)}^{2}}$$

(2) $$∆E^{*}=\sqrt{{\left(L_{2}^{*}-L_{1}^{*}\right)}^{2}+{\left(a_{2}^{*}-a_{1}^{*}\right)}^{2}+{\left(b_{2}^{*}-b_{1}^{*}\right)}^{2}}\;$$

(3) $$∆L^{*}=L_{2\left(\mathrm{aged}\;\mathrm{specimen}\right)}^{*}-L_{1\left(\mathrm{unaged}\;\mathrm{specimen}\right)}^{*}\;$$

(4) $$∆a^{*}=a_{2\left(\mathrm{aged}\;\mathrm{specimen}\right)}^{*}-a_{1\left(\mathrm{unaged}\;\mathrm{specimen}\right)}^{*}\;$$

(5) $$∆b^{*}=b_{2\left(\mathrm{aged}\;\mathrm{specimen}\right)}^{*}-b_{1\left(\mathrm{unaged}\;\mathrm{specimen}\right)}^{*}$$

where ΔL*, Δa*, and Δb* are differences in L*, a*, and b* values between unaged samples color and aged samples color. Total color change (∆E*) values calculated after aging are given in Table 6.

The color of the yellow samples at all infill ratios became matte after the salt spray test. The highest color change was observed in the yellow samples with a 50% infill ratio, while the lowest color change occurred in the blue samples with a 100% infill ratio. For all colors, the total color change values in samples with a 100% infill ratio were lower compared to those with 50% and 75% infill ratios. This difference is attributed to the void-free structure of materials with a 100% infill ratio [57].

## 4. Conclusions

This study investigated the effect of aging on the color and mechanical properties of 3D-printed ABS+ materials with different colors (yellow, green, and blue) and infill ratios (100%, 75%, and 50%) using a salt spray test. It was found that the mechanical properties of ABS+ samples varied depending on both color and infill ratio when exposed to saline environments for extended periods. Therefore, both color and infill ratio should be considered when selecting plastics, particularly for applications in the maritime industry.

Mechanical properties were observed to vary with color across all infill ratios, although the yellow and green samples exhibited relatively similar performance. The highest ultimate tensile strength (UTS) values were recorded as 18.98 MPa in the yellow sample at 50% infill ratio, and 22.35 MPa and 35.85 MPa in the green samples at 75% and 100% infill ratios, respectively. The highest flexural strengths across all infill ratios were also observed in the green samples, measuring 34.25 MPa, 40.61 MPa, and 60.35 MPa, respectively. For applications requiring high mechanical strength, 3D-printed components should be fabricated with 100% infill ratio.

The salt spray test had a more pronounced effect on ABS+ samples with lower infill ratios, with tensile strength decreasing for all colors at 50% infill ratio. Among aged samples, the highest UTS value was 33.46 MPa, observed in the green sample at 100% infill ratio. An increase in tensile strength was observed in blue samples at 75% and 100% infill ratios (unaged: 20.34 MPa and 27.18 MPa; aged: 20.84 MPa and 28.11 MPa). No significant changes were detected in flexural strength after aging. Overall, the yellow and green samples were more affected by aging at lower infill ratios.

After aging, tensile strength decreased by 65.53% in red samples at 20% infill ratio, and by 78.9% in orange samples at 60% infill ratio. At 100% infill ratio, the yellow samples showed a 6.9% increase in tensile strength, whereas the orange and red samples exhibited decreases of 3.16% and 21.27%, respectively. At 100% infill, the orange samples were less affected by aging, while the red samples were more significantly impacted. The increase in tensile strength observed in yellow samples after aging may be attributed to the reflection of a large portion of incident light. The most pronounced color change following the salt spray test was observed in the yellow samples. Aging had a more significant impact on the color of samples with 50% and 75% infill ratios due to the presence of voids.

After the salt spray test, an increase in surface roughness was observed in the samples. This increase can be attributed to the abrasive effect of the prepared sodium chloride solution.

Although ABS materials produced via additive manufacturing demonstrate considerable potential for marine applications, their widespread commercial adoption remains limited. The ability to fabricate marine components through 3D printing not only offers time and cost efficiencies but also presents significant opportunities to address existing gaps in the industry. Furthermore, ongoing research in this field is expected to guide future policies and studies that may redefine the role of additive manufacturing in maritime applications [58].

A longstanding challenge in additive manufacturing research involves the proprietary formulations developed by filament manufacturers. Despite this limitation, the use of commercial filaments—such as the eSUN ABS+ filaments utilized in this study—has become a common practice. While production processes remain consistent across different samples, variations are primarily due to differences in filament color. This study employed optimized processing parameters to systematically evaluate the potential of 3D-printed materials for marine industry applications. Nonetheless, further research is necessary to understand how the performance of ABS+ materials evolves over time in marine environments.

## Figures and Tables

**Table 1:** Properties of test materials [30].

ABS+	
Density (g/cm^3^)	1.06
Tensile Strength (MPa)	40
Elongation at Break (%)	30
Flexural Strength (MPa)	68
Flexural Modulus (MPa)	1203

**Table 2:** Tensile strength and % elongation values of unaged materials.

Infill Ratio	50%		75%		100%	
Color	UTS (MPa)	Strain (%)	UTS (MPa)	Strain (%)	UTS (MPa)	Strain (%)
Yellow	18.98 ± 0.50	4.1	22.01 ± 0.83	3.3	33.20 ± 1.57	6.9
Green	18.93 ± 1.74	3.4	22.35 ± 0.91	3.2	35.85 ± 0.51	6.1
Blue	18.11 ± 0.51	4.1	20.34 ± 1.78	3.5	27.18 ± 1.87	4.0

**Table 3:** Force and displacement values of unaged materials.

Infill Ratio	50%		75%		100%	
Color	Force (N)	Disp. (mm)	Force (N)	Disp. (mm)	Force (N)	Disp. (mm)
Yellow	31.70 ± 1.42	14.42	38.23 ± 1.05	15.27	58.37 ± 1.16	29.98
Green	34.25 ± 1.56	15.70	40.61 ± 0.64	15.92	60.35 ± 0.54	25.15
Blue	31.88 ± 1.95	17.33	35.85 ± 1.29	14.95	53.60 ± 2.34	25.04

**Table 4:** Tensile strength and % elongation values of aged materials.

Infill Ratio	50%		75%		100%	
Color	UTS (MPa)	Strain (%)	UTS (MPa)	Strain (%)	UTS (MPa)	Strain (%)
Yellow	15.38 ± 0.84	3.6	18.3 ± 1.03	3.1	32.2 ± 0.47	8.8
Green	16.89 ± 1.25	3.3	17.9 ± 1.09	2.8	33.46 ± 1.37	8.6
Blue	15.06 ± 0.39	3.3	20.84 ± 0.51	2.8	28.1 ± 0.27	6.2

**Table 5:** Force and displacement values of aged materials.

Infill Ratio	50%		75%		100%	
Color	Force (N)	Disp. (mm)	Force (N)	Disp. (mm)	Force (N)	Disp. (mm)
Yellow	31.19 ± 0.89	14.18	36.78 ± 1.31	15.29	57.56 ± 1.09	32.10
Green	35.52 ± 0.91	16.62	39.66 ± 0.82	15.79	60.74 ± 0.45	29.98
Blue	30.72 ± 0.77	17.44	34.51 ± 0.97	14.56	53.29 ± 0.39	23.87

**Table 6:** Color values of unaged and aged samples with different colors and infill ratios.

Color		Yellow			Green			Blue		
Infill Ratio		50%	75%	100%	50%	75%	100%	50%	75%	100%
Unaged materials	L_1_*	80.67	81.55	81.55	51.46	51.47	51.00	39.90	39.38	39.34
	a_1_*	0.39	1.52	2.21	−38.18	−37.80	−37.45	−10.62	−10.59	−10.83
	b_1_*	75.13	76.92	76.57	5.70	5.37	5.12	−26.99	−27.12	−27.35
Aged materials	L_2_*	80.73	81.42	81.67	51.50	51.10	50.97	39.59	39.37	39.38
	a_2_*	0.39	1.76	2.25	−38.56	−38.14	−37.79	−10.93	−10.65	−10.83
	b_2_*	76.33	77.55	77.11	5.89	5.62	5.34	−26.79	−26.90	−27.13
ΔE*		1.202	0.683	0.561	0.426	0.565	0.396	0.483	0.228	0.223

## Data Availability

The original contributions presented in this study are included in the article. Further inquiries can be directed to the corresponding author. Conflicts of interest: The authors declare no conflicts of interest.
